# Primitive Tumour of the Pulmonary Valve: Discussion of the Differential Diagnosis

**DOI:** 10.1155/2017/6263578

**Published:** 2017-02-19

**Authors:** A. Hannecart, D. Ndjekembo-Shango, F. Vallot, O. Simonet, M. De Kock

**Affiliations:** ICU Department, Centre Hospitalier Wallonie-Picarde, Tournai, Belgium

## Abstract

There is a paucity of information concerning cardiac tumours of the pulmonary valve due to their rarity at this location. We report a case of a 47-year-old patient suffering from haemoptysis, asthenia, and acute kidney injury (AKI). A transthoracic echocardiography (TTE) revealed a mass on the pulmonary valve. Further diagnostic investigation was completed until he exhibited worsening hemodynamic instability. This case emphasizes the lack of information regarding the management of a pulmonary valve tumour.

## 1. Case Presentation

A 47-year-old man was admitted to the ICU department for haemoptysis, asthenia, and acute kidney injury (AKI). His medical history was significant for chronic alcoholic pancreatitis and epidermoid carcinoma of the tongue. The patient was treated for three acute episodes of pancreatitis in 2008 and 2013, complicated with pancreatic-pleural fistula in 2008 and a thrombosis of the mesenteric-splenic truncus in 2009. In 2008, a transthoracic echocardiography (TTE) was performed showing no valve stenosis or insufficiency. The left ventricle ejection fraction (LVEF) was estimated at 60% with septal hypokinesia. No signs of pulmonary hypertension were noted. In 2011, he was diagnosed with differentiated epidermoid carcinoma of the tongue, treated with chemotherapy and radiation therapy. He suffered AKI due to the toxicity of the platinum-based chemotherapy.

**Figure 1:**
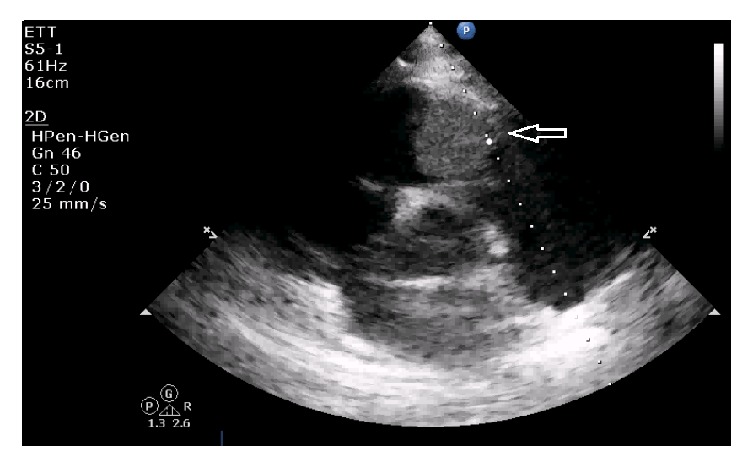
TTE revealing the mass on the pulmonary valve.

**Figure 2:**
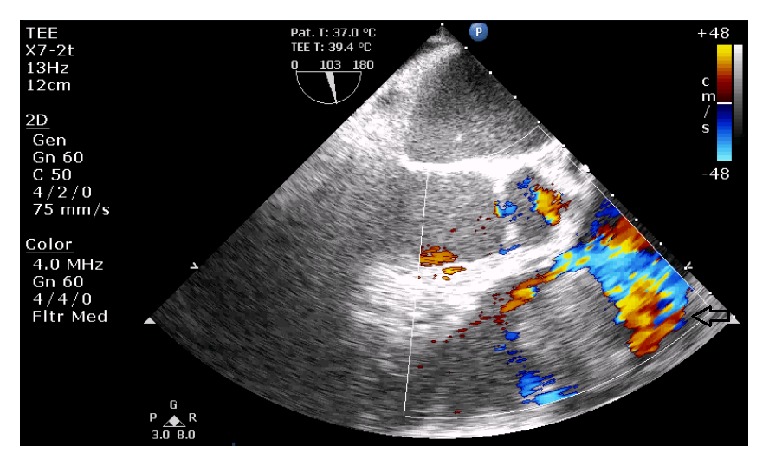
With the TEE on Doppler mode, we can estimate the tumour's blood flow obstruction in the pulmonary artery.

**Figure 3:**
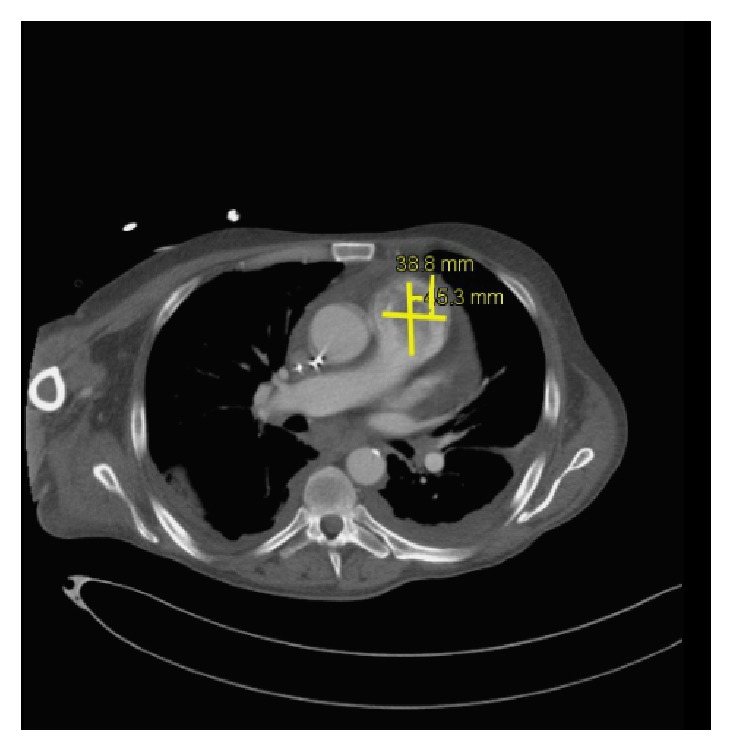
On the CT scan, the tumour is evaluated at 38.8 mm × 43.5 mm.

**Figure 4:**
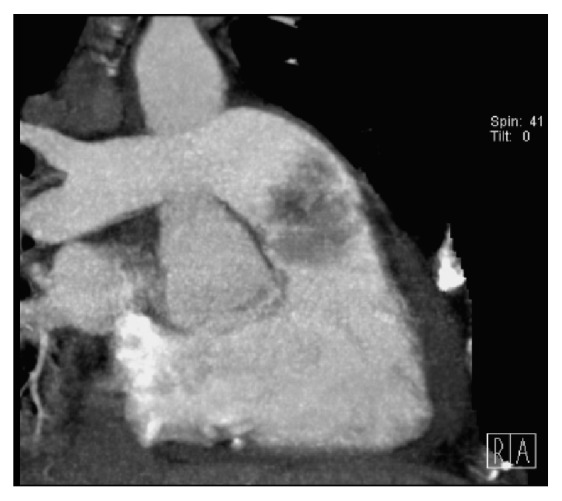
Illustration of the tumour's heterogeneity in contrast uptake.

Within 3 days in the ICU, his platelet level dropped at 10.000/mm^3^, with poor response to transfusion. At day 3, persistent oliguria and hyponatremia led us to start continuous venovenous hemofiltration (CVVH). Repeated pulmonary fiberscopies showed no active bleeding. A gastroscopy excluded any bleeding or significant abnormality of the oesophageal-gastric-duodenal tract. At day 4, TTE revealed signs of pulmonary hypertension (systolic PAP at 50 mmHg + CVP) and a mass (2 × 3 cm) located on the pulmonary valve, causing moderate pulmonary valve insufficiency estimated at 1.5/4 (Figure 1). A thoracic computed tomography (CT) confirmed the tumoural nature of the mass excluding a thrombus formation, on the pulmonary valve. It also revealed the presence of a noncompressive pericardial effusion (Figures 3 and 4). Regarding the thrombocytopenia, a bone marrow biopsy was performed. Along with the immunomarkers, it showed an active inflammatory reactive process with signs of dysmorphia in the megakaryocytic lineage. At day 15, conventional haemodialysis was initiated considering the persistent anuria. Massive haemoptysis occurred along with transient oxygen desaturation. A circulatory collapse requiring IV adrenaline and tracheal intubation happened during fiberscopy. At this moment, a new (TEE) showed that mass on the pulmonary valve almost completely obstructed the blood flow in the pulmonary artery (Figure 2).

No other cardiac abnormality was noted. This episode was followed by alternation of atrial fibrillation (AF) with rapid ventricular response and hypotension and consequently circulatory collapse with oxygen desaturation. This ultimately led to the patient's death. Unfortunately no heart autopsy was performed.

## 2. Diagnostic Evaluation

### 2.1. Imaging Studies Are Essential for the Diagnosis of Cardiac Tumours

Echocardiography is the initial imaging tool to provide information on the morphologic appearance, location, and motion of cardiac masses [1, 2]. A tumour can theoretically always be detected by echocardiography. Therefore TTE is the most readily available noninvasive imaging technique and remains the first-line diagnostic test when a cardiac tumour is suspected [3]. TEE can offer additional imaging planes for further assessment of a lesion like the hemodynamic impact of the tumour on the heart. It is a widely available method and the simplest technique but the quality of the results is operator dependent.

The second-line diagnostic modality is a cardiac CT. It gives us an improved view of the cardiac structures and with contrast enhancement we have a better understanding of the tumour's vascularity and the presence of calcifications [3].

The combination of those imaging techniques is useful to differentiate thrombus from tumour when the lesion appears to arise from a cardiac valve.

The PET scan is proven useful for patients with metastatic tumours, to encounter a possible cardiac involvement. It is not recommended in the initial check-up.

The cardiac MRI remains the best imaging tool to evaluate a cardiac tumour. It can be used to predict its malignancy suspicion with the administration of gadolinium [4]. The initial information of the tumour is vital for it may provide indication of the type of tumour which will evidently influence the treatment management [3]. When compared to CT, MRI has better image resolution and additional tissue characterisation; moreover MRI does not have the attendant risk of radiation.

Transvenous biopsy should be carefully considered knowing the risk of embolization of certain tumours, even benign ones. And in most cases a definitive histological diagnosis is not needed to conduct treatment guidelines. Clinical decisions must be individualised [5].

## 3. Discussion

Because no autopsy was performed on our patient and looking back at his medical history, we have established the most probable differential diagnosis based on the imaging studies of his heart tumour.

We will focus on the primary cardiac tumours, and amongst these, the papillary fibroelastoma (PFE), being the most common tumour [6], and the secondary cardiac tumours considering that the metastatic involvement of the heart is over 20 times more common [4, 7].

Cardiac symptoms can vary from embolization, Cardiac symptoms can vary from embolization and obstruction of blood circulation to sudden death. Interference with the heart valves or direct invasion of the myocardium can also cause arrhythmia, heart block, or pericardial effusion [6, 8].

## 4. Primary Cardiac Tumours

PFEs represent up to 90% of heart valve tumours [5, 9, 10]. They can affect all cardiac valves with a preference for the aortic valve (29%). The pulmonary valve is altered in 13% of the cases [11]. The number of reported cases with PFE on the right side of the heart is extremely low.

Though they are of benign nature and rarely cause valvular dysfunction, they can cause serious complications such as systemic, coronary, and pulmonary embolism [6, 9, 10, 12].

Clinically they can mimic an infectious endocarditis. Although our patient had not previously suffered from pulmonary embolism, his symptoms (haemoptysis) might be explained with possible chronic pulmonary microembolization from the tumour.

## 5. Secondary Cardiac Tumours

Metastasis to the heart is not that infrequent but endocardial and intracavitary metastases are rare, making up 3% to 5% of all cardiac metastases [4]. The most frequently incriminated cancers are the lung, breast, and haematological malignancies along with pleural mesothelioma, melanoma, and ovarian, gastric, renal, hepatocellular, and pancreatic carcinomas [4, 7]. Considering our patient medical history, this diagnosis cannot be excluded.

## 6. Treatment Options

Due to the potential risk of embolization, which can reach up to 25% within 3 years, preoperative anticoagulation and urgent surgical resection is recommended [11, 13]. For asymptomatic patients with nonmobile primary heart tumours, some prefer a close follow-up with periodic clinical evaluation and echocardiography [14].

In most cases a complete resection of the tumour is possible with integrity of the heart valves. But the surgical treatment of a primary cardiac valve tumour is not possible in every hospital, as it requires a specialised cardiac surgery department. However when possible, the prognosis is excellent and no recrudescence has been reported [15].

The use of long-term anticoagulants can be advocated for elderly patients with contraindications for surgery [16].

And, facing a secondary heart tumour, systemic chemotherapy is indicated. Unfortunately the prognosis is limited in localised malignant diseases [17].

## 7. Conclusion

Tumours of the heart cause a symptomatology mostly according to their location rather than by their histopathology. The potential for life-threatening complications recommended that cardiac valve tumours should be managed surgically [6].

Obviously, our patient suffered an obstructive cardiac shock due to the mass obstruction itself or to massive pulmonic tumour embolization. To precisely know the exact nature of the tumour would not have helped us with the treatment. Only, an emergency surgery would have been the only option but the patient was too unstable to undergo such a procedure.

The majority of information found on pulmonary valve tumours is compilation of case reports [6]. There is not enough information to establish guidelines for management. Our patient should have been transferred to a cardiac surgery centre after the first discovery of the pulmonary mass. Our hospital does not cover cardiac procedures. An earlier transfer may have led to surgery in stable hemodynamic conditions and maybe a different outcome.
